# Using Interpretable Artificial Intelligence Algorithms in the Management of Blunt Splenic Trauma: Applications of Optimal Policy Trees as a Treatment Prescription Aid to Improve Patient Mortality

**DOI:** 10.3390/bioengineering12040336

**Published:** 2025-03-24

**Authors:** Vahe S. Panossian, Yu Ma, Bolin Song, Jefferson A. Proaño-Zamudio, Veerle P. C. van Zon, Ikemsinachi C. Nzenwa, Azadeh Tabari, George C. Velmahos, Haytham M. A. Kaafarani, Dimitris Bertsimas, Dania Daye

**Affiliations:** 1 Division of Trauma, Emergency Surgery, Surgical Critical Care, Massachusetts General Hospital, Harvard Medical School, Boston, MA 02114, USA; 2 Operations Research Center, Massachusetts Institute of Technology, Cambridge, MA 02139, USAbolin456@mit.edu (B.S.);; 3 Department of Radiology, Massachusetts General Hospital, Harvard Medical School, Boston, MA 02114, USA; 4 Sloan School of Management, Massachusetts Institute of Technology, Cambridge, MA 02139, USA

**Keywords:** trauma, artificial intelligence, spleen, data-driven decision making, personalized medicine

## Abstract

Background: The identification of the optimal management for blunt splenic trauma—angioembolization (AE), splenectomy, or observation—remains a challenge. This study applies Optimal Policy Trees (OPT), an artificial intelligence (AI) model, to prescribe appropriate management and improve in-hospital mortality. Methods: OPTs were trained on patients with blunt splenic injuries in the ACS-TQIP 2013–2019 to prescribe one of the three interventions: splenectomy, angioembolization (AE), or observation. Prescriptive trees were derived in two separate patient cohorts: those who presented with a systolic blood pressure (SBP) < 70 mmHg and those with an SBP ≥ 70 mmHg. Splenic injury severity was graded using the American Association of Surgical Trauma (AAST) grading scale. Counterfactual estimation was used to predict the effects of interventions on overall in-hospital mortality. Results: Among 54,345 patients, 3.1% underwent splenic AE, 13.1% splenectomy, and 83.8% were managed with observation. In patients with SBP < 70 mmHg, AE was recommended for shock index (SI) < 1.5 or without transfusion, while splenectomy was indicated for SI ≥ 1.5 with transfusion. For patients with SBP ≥ 70 mmHg, AE was recommended for AAST grades 4–5, or grades 1–3 with SI ≥ 1.2; observation was recommended for grades 1–3 with SI < 1.2. Predicted mortality using OPT-prescribed treatments was 18.4% for SBP < 70 mmHg and 4.97% for SBP ≥ 70 mmHg, compared to observed rates of 36.46% and 7.60%, respectively. Conclusions: Interpretable AI models may serve as a decision aid to improve mortality in patients presenting with a blunt splenic injury. Our data-driven prescriptive OPT models may aid in prescribing the appropriate management in this patient cohort based on their characteristics.

## 1. Introduction

Splenic injuries are the most common solid organ injury present in patients with traumatic injuries to the abdomen [1,2]. In recent decades, angioembolization has emerged as an effective non-operative alternative to splenectomy for treating blunt splenic injuries.

Although open surgery is typically the standard treatment for hemodynamically unstable patients with blunt splenic injuries, splenic artery embolization has been reported to be equally effective and safe for controlling hemorrhage, with the added benefit of potentially preserving the spleen [2,3,4,5]. Splenic angioembolisation is primarily used in managing hemodynamically stable patients with American Association of Surgical Trauma (AAST) grade IV and V injuries, with a debatable role in grade III injuries [6,7,8,9].

Despite the demonstrated utility of angioembolization, the challenge for the practicing clinician lies in successfully identifying the patients who would derive the greatest benefit from angioembolization. Another consideration is distinguishing these candidates from those who require a splenectomy.

To this end, prescriptive analytics remain an underutilized resource for deriving data-driven and patient-specific recommendations that maximize improved outcomes. Specifically, prescriptive analytics differ from predictive models in that predictive models primarily focus on grouping patients into risk categories, whereas prescriptive analytics aim to directly determine the best intervention strategy. Optimal Policy Trees have been shown to be a robust tool for data-driven recommendations [10], in addition to being used in predictive models [11]. However, they have not been used in the management of blunt splenic trauma.

In this study, we used a nationwide trauma database to derive and validate a prescriptive machine-learning model designed to optimize the treatment outcomes for blunt splenic injuries. The objective is to offer a data-driven algorithm that guides treatment selection using variables commonly accessible to clinicians in the trauma bay. We hypothesized that the treatment options prescribed by our model may result in a clinically significant improvement over the currently observed mortality.

## 2. Methods

### 2.1. Data Source

We used the American College of Surgeons Trauma Quality Improvement Program (ACS-TQIP) dataset retrospectively spanning 2013 to 2019. This database collects patient characteristics, trauma center characteristics, and hospital events (procedures, complications, discharge disposition, among others) from more than 800 trauma centers all over the United States. Data are collected by trained trauma abstractors through chart review, and the data are routinely audited to ensure quality [12].

### 2.2. Patient Selection

We included patients who were 18 years or older who had blunt splenic injuries. Patients who had no signs of life upon arrival at the emergency department (ED), those who died in the ED, and patients who were transferred to or from another hospital were excluded. Furthermore, patients with missing data on age and length of hospital stay were excluded.

### 2.3. Input Parameters and Outcome Definitions

Parameters included in the model were baseline demographics and characteristics (age, sex, body mass index [BMI]); vitals upon admission (systolic blood pressure [SBP], heart rate [HR], respiratory rate [RR]); Glasgow Coma Scale upon arrival to the emergency department (ED); intubation in the ED; teaching status and the American College of Surgeons (ACS) level of the center; comorbidities; transfusion of packed red blood cells (pRBC) or whole blood within 1 h of arrival; and injuries defined using Abbreviated Injury Scale (AIS) codes. Injury parameters included splenic injury grade (I–V), traumatic brain injury (TBI), pelvic fracture, and presence of kidney, liver, small bowel, colon, and spine injuries. TBI was defined as AIS head severity of 3 or more. Splenic injury grade was classified based on the American Association of Surgical Trauma (AAST) Splenic Grading Scale.

Comorbidities included: Bleeding Disorder, Congestive Heart Failure (CHF), Smoker, Chronic Kidney Disease (CKD), Cerebrovascular Accident (CVA), Diabetes Mellitus, Myocardial Infarction (MI), Peripheral Arterial Disease (PAD), Hypertension (HTN), Chronic Obstructive Pulmonary Disease (COPD), Steroid Use, Cirrhosis.

Interventions were classified using ICD-9 and ICD-10 procedure codes into three categories: splenic angioembolization (AE), splenectomy, and observation.

The main outcome was in-hospital overall mortality until discharge. Post-discharge mortality was not analyzed as it is not collected by the ACS-TQIP database.

### 2.4. Counterfactual Estimation

Counterfactual estimation is a widely used statistical model used to evaluate the potential outcomes and causal effects of medical interventions or treatments using only the available observational data. It involves creating a hypothetical scenario, or “counterfactual”, in which the treatment or intervention is not present and comparing this scenario to the actual outcome observed in the real world where the treatment was applied. By comparing the outcome in the counterfactual scenario to the observed outcome, counterfactual estimation attempts to estimate what would have happened to the outcome had the treatment or intervention not been applied, or vice versa. This can be used to predict the effects of treatments or interventions on patients or populations and to compare the outcomes of different treatments [13].

Since our data set included outcomes only for the observed treatment, the first step in our prescriptive model was counterfactual estimation: utilizing the observed data to infer the unobserved outcomes so that proposed prescription policies could be evaluated. We used a doubly robust reward estimation technique [14,15] that combines an outcome estimator and a propensity estimator [16], where the first aims to estimate the potential outcome given the individual patient characteristics and treatment prescribed, and the second helps adjust and correct for potential treatment assignment confounding bias. We used Random Forest for both estimators, where the outcome estimator concretely predicts the mortality risk under each treatment, and the propensity estimator predicts the probability that a sample is assigned to each of the three treatments. In order to keep the testing set unbiased, separate counterfactual estimators were independently fit to the training and testing sets.

### 2.5. Optimal Policy Tree

Given the estimated counterfactuals, we used Optimal Policy Trees (OPT) to learn an optimal prescription policy that minimizes the post-operative overall mortality rate. OPT is a single-tree-based model that aims at splitting the input patient cohort until each subgroup is as homogenous and separable as possible. The single-tree-based methodology offers the important advantage of interpretability, since every decision the model takes to arrive at its final treatment policy is clearly documented as a binary split of the patient’s characteristics. OPTs have been shown to outperform other prescriptive methodologies [17].

We first split the original dataset into 50% training and 50% testing data across the three dimensions of important components: independent features, outcomes, and treatment. The choice to keep 50% of the data in the testing set, which is more of the data than the usual machine learning practice, was made to ensure we could make a high-quality reward estimation in the test set. We ensured that the result is reproducible by imposing a fixed random seed across all of our experiments. OPTs were then trained on the training data by specifying the types of fitting constraints. Specifically, in order to combat overfitting, the OPTs were trained by restricting each leaf to at least 20 samples, thus ensuring the model did not become overly sensitive to outlier samples and improving its generalizability. Moreover, a grid search was used to tune both the complexity parameter as well as the maximum tree depth (ranging from 3 to 8), which appropriately penalizes overly complicated trees. Once the optimal learner was found, we used the testing set to compute the estimated outcome under this OPT. An example implementation of the OPT model can be found in the Supplemental Materials, and the OPT model can be accessed on its hosting website, Interpretable AI. The programming language of Python Version 3.13.2, R, or Julia can be used to implement the model.

Each colored node in the tree indicates a leaf, which consists of the patients in the training set that were assigned to that particular sub-cohort, along with their respective sample size. The more intense the color, the bigger the difference between the optimal treatment (the one it prescribed) and the sub-optimal treatment.

### 2.6. Ethical Oversight

Due to the de-identified nature of the dataset, this study was approved as exempt by the Mass General Brigham Institutional Review Board. We adhered to the Transparent reporting of a multivariable prediction model for individual prognosis or diagnosis (TRIPOD) reporting guidelines.

## 3. Results

A total of 54,345 patients were included. A total of 3.1% received splenic AE, 13.1% splenectomy, and 83.8% observation. Table 1 describes the baseline patient characteristics and demographics.

The median age was 38 years (IQR: 26–56), with 33.6% being females. Upon arrival, 10.1% required intubation in the ED, and half of the participants were admitted to a university hospital.

Splenic injuries were classified as grade 4 in 17.4% of cases and grade 5 in 7.9% of cases. Overall mortality was 8.3%, with significant differences among the three groups: 9.4% in the splenic AE group, 20.0% in the splenectomy group, and 6.5% in the observation group (*p* < 0.001).

Those undergoing splenectomy had a higher incidence of grade 5 splenic injury compared to the splenic AE and observation groups (28.5% vs. 15.1% and 4.4%, respectively, *p* < 0.001). Moreover, they also had a greater prevalence of concomitant liver injury (30.9% vs. 21.7% and 20.3%, *p* < 0.001) and kidney injury (17.6% vs. 16.2% and 13.3%, *p* < 0.001).

Furthermore, 6.9% of the splenectomy group presented with an SBP upon arrival of less than 70 mmHg, compared to 5.4% in the splenic AE group and 1.8% in the observation group (*p* < 0.001).

### 3.1. OPT and Splenic AE in SBP < 70 mmHg

**Figure 1:**
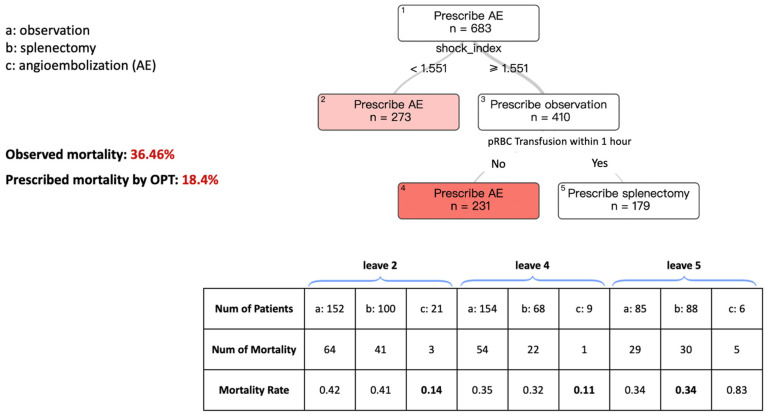
Optimal Policy Tree (OPT), which prescribes splenic AE vs. splenectomy or observation to improve in-hospital mortality in patients with SBP < 70 mmHg. The prescribed mortality is lower than the mortality observed in the dataset.

Figure 1 describes the model for splenic AE, splenectomy, or observation, aiming to improve mortality in patients presenting with an SBP of less than 70 mmHg.

The tree had transparency and interpretability, with a relatively concise set of decision branches. Each rectangular box symbolizes a “leaf”, with the data point used by the model at each branch point listed beneath it. Within these leaves, the model recommends either “Prescribe Splenic AE”, “Prescribe Splenectomy”, or “Prescribe Observation” as the treatment. The number of patients (N) on each leaf is also provided. The color of the terminal leaves corresponds to each treatment and indicates the strength of the prescription. Darker colors signify a more confident prescription, while lighter colors suggest a less pronounced difference between the choice to prescribe the different intervention types.

Starting from the top of the tree, at Leaf #1, we see the recommendation to “Prescribe AE” as having a patient count of N = 683. The first decision point in the branching asks about the shock index, which is calculated by dividing the heart rate by the SBP upon presentation. If the shock index is ≥1.551, Leaf #3 advises “Prescribe Observation”. This indicates the model’s suggestion that these patients are observed, but for a more assured prescription, we need to move further down the tree.

Moving to the next leaf point, which asks for the transfusion of packed red blood cells (pRBC) within one hour, if the answer is No, the tree recommends “Prescribe AE” Conversely, if the answer is Yes, the recommendation shifts to splenectomy.

Returning to Leaf #1, following the branch to the left for patients with a shock index <1.551 leads to Leaf #2, which recommends AE for these cases. Consequently, the terminal leaflet represents the final prescription of the model for each pathway within the tree. In this patient cohort, with SBP < 70 mmHg on presentation, the predicted mortality rate is 18.4% when using the prescribed treatment based on our algorithms compared to the observed mortality rate of 36.46% based on current practice patterns.

The table below the figure displays the observed mortality rates for patients in the terminal leaves across different treatment groups based on the original dataset. The bolded mortality rate represents the treatment associated with the lowest observed mortality, which corresponds to the recommendations of the OPT model. For example, in leaf 2, following the OPT branching process, there were 273 patients in total. Of these, 21 patients received angioembolization, with an observed mortality rate of 0.14—the lowest among the three treatment modalities, consistent with the OPT recommendation.

### 3.2. OPT and Splenic AE in SBP ≥ 70 mmHg

**Figure 2:**
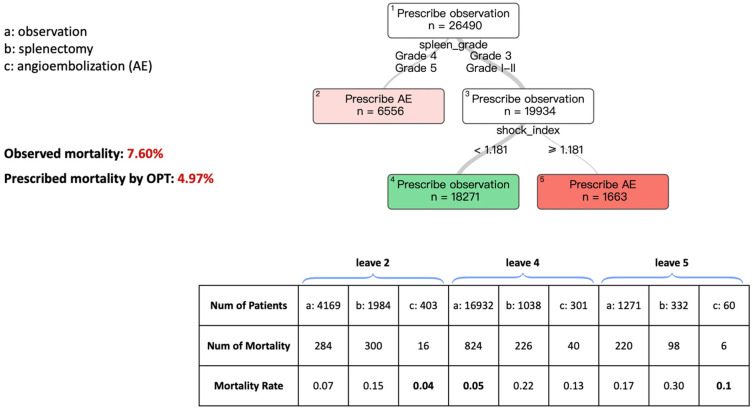
Optimal Policy Tree (OPT) that prescribes plenic AE vs. splenectomy or observation to improve in-hospital mortality in patients with SBP ≥ 70 mmHg. The prescribed mortality is lower than the mortality observed in the dataset.

Figure 2 describes the OPT model for patients presenting with an SBP of ≥ 70 mmHg. Starting from the top of the tree, Leaf #1 recommends observation with n = 26,490. However, for a more accurate prescription, it asks for the splenic injury grade. If it is a grade 4 or 5 injury, AE is prescribed in Leaf #2. However, if it is a splenic grade 1–2 or grade 3 injury, observation is prescribed. To obtain a more accurate prescription for these patients, the next question refers to the shock index. In patients with a shock index <1.181, the model prescribes observation, whereas AE is prescribed in those with a shock index of ≥1.181. In this subset of patients, the predicted mortality rate is 4.97% when using the prescribed treatment based on our algorithms, compared to the observed mortality rate of 7.60% based on current practice patterns.

## 4. Discussion

In this study, we aimed to investigate the role of Optimal Policy Trees (OPT) in guiding clinical decision-making for patients with blunt splenic injuries, with the ultimate goal of improving mortality outcomes in this high-risk population. The OPT model prescription resulted in reduced hospital mortality in patients with blunt splenic injuries. In patients with an SBP < 70 mmHg upon presentation, mortality rates went from 36.46% to 18.4%, and in patients with SBP ≥ 70, the mortality went from 7.60% to 4.97%.

In a recent study by Guinto et al. (2020) [18], a retrospective analysis was conducted on 1052 patients with an initial SBP ≤ 90 mmHg and an AIS score of 4 or 5, utilizing a national trauma database. Nearly all patients (95%) underwent splenectomy, while a small minority received splenic angioembolization (5%). The mortality rates were 29.7% for embolization and 18% for splenectomy. When compared to mortality rates in our OPT model, the figures are nearly identical for the splenectomy subgroup. However, our model reveals a significant variance in the utilization of embolization in hemodynamically unstable patients. In the optimized model for blunt splenic trauma management, embolization was employed much more frequently compared to the aforementioned study (74% vs. 5%). Consequently, our model suggests that the mortality rate in hemodynamically unstable patients undergoing splenic AE is lower than that reported in the aforementioned study. When clinically appropriate, previous studies have demonstrated that early arterial embolization in intra-abdominal solid organ injuries is associated with improved outcomes, including reduced hospital stays, lower transfusion requirements, and decreased overall costs, emphasizing the importance of timely intervention in patients with splenic trauma [19].

The current guidelines do not recommend treating hemodynamically unstable patients with splenic AE [20]. Instead, it is advised that all hemodynamically unstable patients undergo immediate laparotomy. However, our optimized model demonstrates that alternative management strategies are viable for severely injured patients. The decision tree recommends splenectomy only if the shock index is ≥1.551 and the patient has received a transfusion of packed red blood cells (pRBC) within one hour. Our findings demonstrate the potential of OPT as a valuable tool in optimizing the management of blunt splenic trauma. By leveraging novel and interpretable AI models, we were able to develop a prescriptive model that offers clinicians clear and data-driven guidance in determining the most appropriate course of action for individual patients. The application of OPT in blunt splenic injury management offers insights into the potential benefits of AI-driven decision support systems across various traumatic conditions.

Advanced machine learning models have demonstrated the ability to accurately predict the need for massive transfusion, failure of nonoperative management, and mortality risk in pediatric blunt solid organ injuries using the clinical, laboratory, and imaging data available within the first few hours of admission, highlighting the potential of AI-driven decision support tools in trauma care [21]. Specifically in splenic trauma, recent advancements in deep learning have demonstrated high accuracy in detecting splenic injuries on CT scans, with AI-driven algorithms capable of localizing and classifying traumatic injuries with strong sensitivity and specificity, suggesting their potential application in trauma triage and decision-making [22]. However, to our knowledge, no previous research exists on using AI-driven algorithms in the management of blunt splenic trauma.

Despite these promising findings, it is important to acknowledge the limitations of our study. The retrospective nature of the data and the potential for selection bias may limit the generalizability of our findings. Also, our study lacked data on critical parameters, such as contrast extravasation on CT scans, haemoperitoneum, the presence of pseudoaneurysm, arteriovenous fistula, and a granular timeline of clinical deterioration and interventions, which could have influenced treatment decisions and outcomes. The database used for this study lacks multiple measurements of data points across time, limiting us to isolated data points, such as SBP only at the time of presentation. Furthermore, the training data were sourced from multiple centers that operate at varying capacities and exhibit inter-center variability in practice patterns and outcomes, potentially introducing bias into the dataset. 

Additionally, our study lacks long-term outcomes of non-operative management, such as delayed splenic rupture or splenic pseudoaneurysm (SPA) formation. The rupture of the SPA may lead to the delayed rupture of the spleen. Delayed SPA can be detected within 15 days after injury onset [23]. The occurrence of delayed spleen rupture has become infrequent yet remains a significant complication of non-operative management. Various studies have noted that 1–2% of patients required hospital readmission due to post-discharge bleeding and subsequently underwent splenectomy [24,25].

Further research is needed to validate the performance of our prescriptive OPT algorithm in prospective studies and to refine its clinical utility using data measured multiple times across time.

## 5. Conclusions

Using interpretable AI algorithm, we derived data-driven prescriptive algorithms to manage blunt splenic trauma. By providing clinicians with clear and data-driven guidance, the OPT algorithm has the potential to be used as a clinical support tool to improve outcomes for patients with blunt splenic injuries. Our AI-driven prescriptive model demonstrated the potential to improve mortality outcomes in patients with blunt splenic injuries. Further validation is warranted to assess their clinical applicability. As AI technologies continue to evolve, there is significant potential for further innovation in trauma care and the development of more advanced decision support systems.

## Figures and Tables

**Table 1:** Baseline demographics and characteristics of the study sample.

	Total	Observation	Splenectomy	Angioembolization	*p*-Value
	N = 54,345	N = 45,546	N = 7128	N = 1671	
Median Age (IQR)	38 (26–56)	38 (25–55)	39 (27–56)	44 (28–59)	<0.001
Sex					<0.001
Male	36,066 (66.4%)	30,061 (66.0%)	4909 (68.9%)	1096 (65.6%)	
Female	18,269 (33.6%)	15,476 (34.0%)	2218 (31.1%)	575 (34.4%)	
Median SBP (IQR)	124 (107–141)	126 (110–142)	110 (90–130)	112 (93–133)	<0.001
ED SBP					<0.001
SBP >= 70 mm Hg	52,217 (97.5%)	44,151 (98.2%)	6510 (93.1%)	1556 (94.6%)	
SBP < 70 mm Hg	1366 (2.5%)	792 (1.8%)	486 (6.9%)	88 (5.4%)	
Median Pulse (IQR)	95 (80–111)	93 (80–109)	103 (85–124)	100 (82–119)	<0.001
Median Respiratory rate (IQR)	19 (16–22)	19 (16–22)	20 (16–24)	20 (17–24)	<0.001
Median Pulse oximetry (IQR)	98 (95–100)	98 (95–100)	97 (94–100)	97 (94–100)	<0.001
Median GCS (IQR)	15 (14–15)	15 (14–15)	14 (6–15)	15 (13–15)	<0.001
Median BMI (IQR)	26.8(23.4–31.4)	26.9 (23.4–31.5)	26.6 (23.1–30.9)	27.1 (23.3–31.9)	<0.001
Intubated in ED	5497 (10.1%)	4069 (8.9%)	1227 (17.2%)	201 (12.0%)	<0.001
Teaching Status					0.96
Community	18,627 (34.4%)	15,628 (34.4%)	2418 (34.1%)	581 (34.9%)	
Nonteaching	7058 (13.0%)	5923 (13.0%)	920 (13.0%)	215 (12.9%)	
University	28,471 (52.6%)	23,846 (52.5%)	3756 (52.9%)	869 (52.2%)	
ACS Verification Level					<0.001
1	25,709 (57.3%)	21,443 (56.6%)	3408 (61.5%)	858 (61.9%)	
2	11,304 (25.2%)	9569 (25.2%)	1374 (24.8%)	361 (26.0%)	
3	7830 (17.5%)	6900 (18.2%)	763 (13.8%)	167 (12.0%)	
Bleeding Disorder	886 (1.6%)	724 (1.6%)	114 (1.6%)	48 (2.9%)	<0.001
CHF	863 (1.6%)	723 (1.6%)	101 (1.4%)	39 (2.3%)	0.027
Smoker	13,774 (25.5%)	11,638 (25.7%)	1762 (25.0%)	374 (22.5%)	0.008
CKD	387 (0.7%)	324 (0.7%)	44 (0.6%)	19 (1.1%)	0.077
CVA	505 (0.9%)	447 (1.0%)	47 (0.7%)	11 (0.7%)	0.017
Diabetes Mellitus	4526 (8.4%)	3847 (8.5%)	506 (7.2%)	173 (10.4%)	<0.001
MI	257 (0.5%)	202 (0.4%)	43 (0.6%)	12 (0.7%)	0.060
Peripheral arterial disease	151 (0.3%)	118 (0.3%)	25 (0.4%)	8 (0.5%)	0.11
Hypertension	10,355 (19.2%)	8741 (19.3%)	1222 (17.3%)	392 (23.6%)	<0.001
COPD	2209 (4.1%)	1863 (4.1%)	283 (4.0%)	63 (3.8%)	0.76
Steroid use	239 (0.4%)	195 (0.4%)	33 (0.5%)	11 (0.7%)	0.36
Cirrhosis	824 (1.5%)	600 (1.3%)	185 (2.6%)	39 (2.3%)	<0.001
Transfusion RBC, 1 h	5272 (9.7%)	2479 (5.4%)	2390 (33.5%)	403 (24.1%)	<0.001
Transfusion, whole blood, 1 h	412 (0.8%)	178 (0.4%)	206 (2.9%)	28 (1.7%)	<0.001
Spleen Grade					<0.001
Grade 1–2	29,434 (54.2%)	27,901 (61.3%)	1208 (16.9%)	325 (19.4%)	
Grade 3	11,182 (20.6%)	8969 (19.7%)	1756 (24.6%)	457 (27.3%)	
Grade 4	9443 (17.4%)	6676 (14.7%)	2131 (29.9%)	636 (38.1%)	
Grade 5	4286 (7.9%)	2000 (4.4%)	2033 (28.5%)	253 (15.1%)	
Liver Injury	11,797 (21.7%)	9233 (20.3%)	2201 (30.9%)	363 (21.7%)	<0.001
Kidney Injury	7593 (14.0%)	6065 (13.3%)	1257 (17.6%)	271 (16.2%)	<0.001
Small Bowel Injury	7593 (14.0%)	6065 (13.3%)	1257 (17.6%)	271 (16.2%)	<0.001
Colon Injury	1066 (2.0%)	855 (1.9%)	204 (2.9%)	7 (0.4%)	<0.001
Spine Injury	3759 (6.9%)	2912 (6.4%)	713 (10.0%)	134 (8.0%)	<0.001
Pelvic Fracture	4027 (7.4%)	3034 (6.7%)	792 (11.1%)	201 (12.0%)	<0.001
TBI	10,949 (20.1%)	8601 (18.9%)	1958 (27.5%)	390 (23.3%)	<0.001
In-hospital Mortality	4536 (8.3%)	2953 (6.5%)	1426 (20.0%)	157 (9.4%)	<0.001

IQR: inter-quartile range; SBP: systolic blood pressure; ED: emergency department; GCS: Glasgow Coma Scale; BMI: body mass index; ACS: American College of Surgeons; CHF: congestive heart failure; CKD: chronic kidney disease; CVA: cerebrovascular accident; MI: myocardial infarction; COPD: chronic obstructive pulmonary disease; RBC: red blood cell; TBI: traumatic brain injury.

## Data Availability

Data for this study is not available due to privacy and ethical restrications on the use of data in this dataset.

## Supplementary Materials

The following supporting information can be downloaded at: https://www.mdpi.com/article/10.3390/bioengineering12040336/s1.
