# Supplementary material for: Using Interpretable Artificial Intelligence Algorithms in the Management of Blunt Splenic Trauma: Applications of Optimal Policy Trees as a Treatment Prescription Aid to Improve Patient Mortality
Source: Bioengineering (Basel). 2025 Mar 24;12(4):336. doi: 10.3390/bioengineering12040336 (PMC12024711; doi:10.3390/bioengineering12040336)
Supplement: Supplementary file 1 [file bioengineering-12-00336-s001.zip › bioengineering-3509649-supplementary.pdf]

Supplement Materials and Analysis

1. Exploratory Data Analysis:

- **Handling Missing Data:** We applied Optimal K-Nearest-Neighbor Imputation (https://docs.interpretable.ai/stable/OptImpute/) to handle missing values. Out of 54,345 patient records, 18,391 contained missing values. A summary of missing values across all features is provided below.

```
inc_key                     0
sex                        10
age                         0
sbp                       762
pulserate                 611
respiratoryrate          1633
pulseoximetry            2132
totalgcs                 1241
intubated                   0
bmi                      6744
teachingstatus            189
verificationlevel        9502
cc_bleeding               358
cc_chf                    352
cc_smoking                282
cc_renal                  356
cc_cva                    361
cc_diabetes               329
cc_mi                     355
cc_pad                    356
cc_hypertension           305
cc_copd                   343
cc_steroid                354
cc_cirrhosis              353
transf_rbc_1hr              0
transf_wholeblood_1hr       0
spleen_grade                0
liver_inj                   0
kidney_inj                  0
smallbowel_inj              0
colon_inj                   0
spine_inj                   0
pelvic_fx                   0
tbi                         0
i_observation               0
i_splenectomy               0
i_ae_spleen                 0
o_mortality                 0
treatment                   0
dtype: int64
```

  - **Why not simply delete missing data?** As seen from 0 missing values of i_obseration, i_splenectomy, i_ae_spleen, the missing values are mainly from deceased people. Simply removing records with missing values would lower the observed mortality rate, introducing bias in OPT's prescription results. A summary of the observed mortality across different treatment groups is provided below.

	Overall mortality	Observation mortality	Splenectomy mortality	Angioembolization mortality
Mortality if deleting missing values	0.061	0.048	0.1349	0.07
Overall mortality	0.0835	0.0643	0.188	0.081

- **Distribution of Variables Around Mean:**
  - Please see attached folder "Variables_Distributions" for reference.

2. Counterfactual Estimation:

- **Random Forest vs. XGBoost as Estimator:**
  - We compared Random Forest and XGBoost using a 50/50 train–test split to evaluate their robustness in counterfactual estimation.
  - We trained an OPT on each train/test split and computed the Average Treatment Effect (ATE).
  - We repeated this for 50 different splits and plotted the distribution of ATE values.
  - Random Forest showed lower variance (narrower spread), indicating greater stability compared to XGBoost.
  - **Conclusion:** Random Forest is a more robust estimator for our dataset.
  - (Refer to the attached slides for detailed visualizations).

Compare classifier RF and XGBoost by boxplots

Prescription Reward

	Test 1	Test 2	Test 3	...	Test 50	
Train 1-OPT	0.0662	0.0693	0.0673		0.0656	→ μ_1, σ_1^2
Train 2-OPT	0.0632	0.0433	0.0820		0.0556	→ μ_2, σ_2^2
Train 3-OPT	0.0693	0.0617	0.0640		0.0628	→ μ_3, σ_3^2
...						
Train 50-OPT	0.0640	0.0597	0.0637		0.0586	→ μ_N, σ_N^2

−

Actual Treatment Reward

	Test 1	Test 2	Test 3	...	Test 50	
Train 1-OPT	0.0925	0.0820	0.0860		0.0862	→ μ_1, σ_1^2
Train 2-OPT	0.0925	0.0820	0.0860		0.0862	→ μ_2, σ_2^2
Train 3-OPT	0.0925	0.0820	0.0860		0.0862	→ μ_3, σ_3^2
...						
Train 50-OPT	0.0925	0.0820	0.0860		0.0862	→ μ_N, σ_N^2

=

Average Treatment Effect (reward)

	Test 1	Test 2	Test 3	...	Test 50	
Train 1-OPT	-0.0262	-0.0126	-0.0187		-0.0206	→ μ_1, σ_1^2
Train 2-OPT	-0.0292	-0.0386	-0.0039		-0.0305	→ μ_2, σ_2^2
Train 3-OPT	-0.0232	-0.0202	-0.0219		-0.0234	→ μ_3, σ_3^2
...						
Train 50-OPT	-0.0284	-0.0222	-0.0222		-0.0275	→ μ_N, σ_N^2

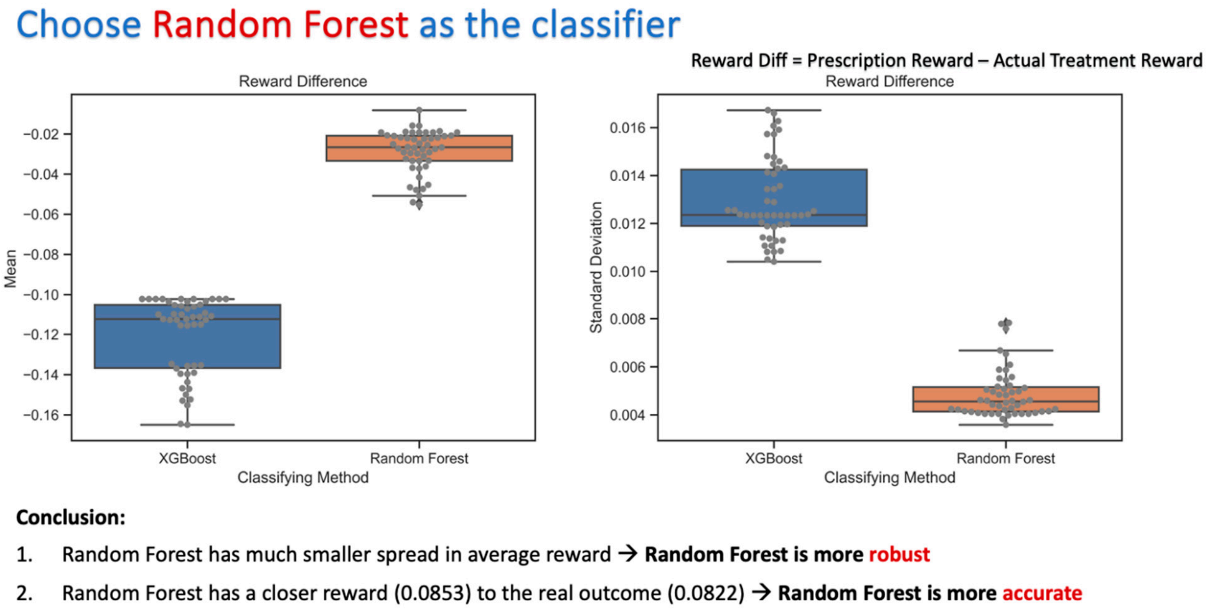

3. Training on OPT:

- **Training Framework: We use Interpretable AI open-source platform: https://docs.interpretable.ai/stable/OptimalTrees/quickstart/policy_categorical/**
- **Training–Test split: 50/50**
- **Hyperparameter Grid:**
    - **minbucket = 20** (we controlled the minbucket to avoid overfit).
    - **max_depth= 3:8** (we controlled the depths to avoid overfit and underfit).
    - We performed a grid search to find the optimal minbucket and max_depth values

(https://docs.interpretable.ai/stable/IAIBase/reference/#IAI.GridSearch).

- **Cross-Validation Consideration from Reviewer 2**

    We did not apply cross-validation for training OPT because the counterfactual estimation must remain consistent between the training and test sets. Some arguments in our paper might need be made on this point.

    (To Yu: Correct me if I was wrong.)

4. Evaluation on OPT:

- **Feature Importance Analysis:**

    - We conducted 5-fold cross-validation using a Random Forest classifier trained on the actual treatments.
    - The dependent variable was the observed treatment (operating on the dataset after imputation).
    - We ranked feature importance to compare how OPT's decision-making process differs from actual clinical practices in treatment selection (please see attached folder "5_fold_classification_model" for reference).

5. Summary on the Workflow:

- Handle Missing Data:
    - Apply Optimal KNN imputation to fill missing values.
- Counterfactual Estimation:
    - Use Doubly Robust estimation for treatment effect estimation on both training and test data.
- Train the OPT:
    - Train OPT based on the estimated reward from the training set.
    - Predict OPT's recommended treatments on the test set.
- Compare OPT vs. Actual Clinical Decisions:
    - Train a Random Forest classifier on real-world treatment data.
    - Rank feature importance and compare with OPT's decision rules. We can also compare with other papers' decision rules and present our contributions.
